# Noise and Breakdown Characterization of SPAD Detectors with Time-Gated Photon-Counting Operation

**DOI:** 10.3390/s21165287

**Published:** 2021-08-05

**Authors:** Hiwa Mahmoudi, Michael Hofbauer, Bernhard Goll, Horst Zimmermann

**Affiliations:** Institute of Electrodynamics, Microwave and Circuit Engineering, Vienna University of Technology, 1040 Vienna, Austria; michael.hofbauer@tuwien.ac.at (M.H.); bernhard.goll@tuwien.ac.at (B.G.); horst.zimmermann@tuwien.ac.at (H.Z.)

**Keywords:** afterpulsing, dark-count, photon-counting, single-photon avalanche diode (SPAD), time-gated operation

## Abstract

Being ready-to-detect over a certain portion of time makes the time-gated single-photon avalanche diode (SPAD) an attractive candidate for low-noise photon-counting applications. A careful SPAD noise and performance characterization, however, is critical to avoid time-consuming experimental optimization and redesign iterations for such applications. Here, we present an extensive empirical study of the breakdown voltage, as well as the dark-count and afterpulsing noise mechanisms for a fully integrated time-gated SPAD detector in 0.35-μm CMOS based on experimental data acquired in a dark condition. An “effective” SPAD breakdown voltage is introduced to enable efficient characterization and modeling of the dark-count and afterpulsing probabilities with respect to the excess bias voltage and the gating duration time. The presented breakdown and noise models will allow for accurate modeling and optimization of SPAD-based detector designs, where the SPAD noise can impose severe trade-offs with speed and sensitivity as is shown via an example.

## 1. Introduction

CMOS realization in array format and high sensitivity in the visible and near-infrared spectral range have made the single-photon avalanche diode (SPAD) a very attractive low-light detector for different sensor and imaging applications such as laser ranging, quantum processing, biomedical microscopy, astronomical telescopes, optical communication, etc. [1,2,3,4,5,6,7]. By exploiting the avalanche mechanism to generate a macroscopic current pulse, when the device is biased above its breakdown voltage (Geiger mode), the absorption of a single photon can generate a detectable count signal with eliminated read noise. Despite numerous advantages, the Geiger mode (digital) operation imposes other noise mechanisms, i.e., SPAD intrinsic parasitics, creating avalanche detection counts without a photon being absorbed. This includes detection counts triggered by thermally or tunneling-generated carriers (i.e., dark-count noise), as well as the counts that are strongly correlated with previous avalanche detections (so-called afterpulsing noise) [8,9]. Afterpulsing is triggered by charge carriers trapped during the previous avalanches and released with a delay when the SPAD is recovered. As different types of traps can contribute to afterpulsing and their domination depends on the device characteristics and even biasing and quenching circuit, it is difficult to provide a universal mathematical model with a physical meaning for afterpulsing; thus, every detector design has to be characterized individually [10].

A careful SPAD noise characterization is critical for the detector design and performance optimization of SPAD-based systems, and therefore, extensive studies have been conducted to characterize, model, and mitigate the SPAD intrinsic parasitic effects [11,12,13,14,15,16]. In Mahmoudi et al. [9], a statistical approach was presented to measure different SPAD noise mechanisms using experimental data acquired in a dark condition, which is the most basic and straightforward performance measurement of the SPAD. This statistical approach, however, is only appropriate for detectors with quench/reset circuitry, where the SPAD bias is returned to above the breakdown after any detection to be ready and waiting for the next detection (i.e., free-running operation). Although the quench/reset operation of SPAD has become the most utilized implementation in many SPAD applications, the time-gated SPAD is a very attractive implementation, especially to further suppress the SPAD intrinsic noise when the noise reduction is extremely critical [17,18,19,20]. As is explained later, the noise characterization of a time-gated SPAD requires a different approach as compared to the free-running quench/reset implementation. Therefore, in this work, we investigate the dark-count and afterpulsing noise mechanisms for a time-gated CMOS SPAD detector based on experimental data acquired in a dark condition. It is shown that the “effective” SPAD breakdown shows a gating-duration dependency, which needs to be taken into account for noise characterization and modeling. The operation principle of the time-gated SPAD and our measurement setup to acquire dark-noise data are explained in Section 2. The breakdown and noise analysis and modeling approaches are described in Section 3. The presented approaches allow for accurate modeling and efficient optimization of SPAD-based detectors with time-gated operation as is shown via an example in Section 4, and finally, the conclusion and future work are summarized in Section 5.

## 2. Operation Principle and Measurement Setup

### 2.1. Time-Gated Detection Concept

**Figure 1:**
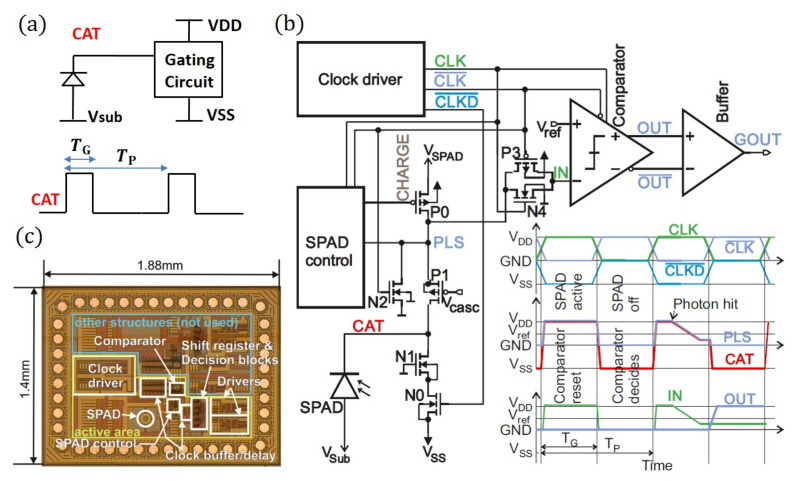
(**a**) Basic concept of the time-gated detection, (**b**) circuit block diagram, and (**c**) microphotograph of the fully integrated time-gated SPAD data receiver test chip [21]. The main supply voltages of the gating circuit are $V_{\mathrm{DD}}=\;$3.3 V and $V_{\mathrm{SS}}=\;-$3.3 V.

In order to decrease the SPAD intrinsic noise, the time-gated operation is considered as an appropriate solution by allowing the SPAD to fire within a short time ($T_{G}$) over a well-defined pulse period ($T_{P}$), as is shown in Figure 1a. Here, the gating circuit applies a periodic signal on the SPAD cathode, and the substrate voltage connected to the SPAD anode is set at a level that the SPAD is biased above its breakdown only during $T_{G}$. Furthermore, the circuit is able to distinguish (count) if an avalanche is triggered during $T_{G}$ using a comparator, which can sense a voltage drop on the SPAD cathode within $T_{G}$.

Outside the gate-ON time $T_{G}$, the SPAD is blind to any detection as the bias is kept below breakdown. This can significantly decrease the SPAD noise, especially the afterpulsing, as the trapped carriers can be released without triggering an afterpulse. In contrast, when the SPAD is operated in a free-running quench/reset mode, the released carriers can trigger afterpulse events any time after the SPAD is reset above breakdown again, and thus, the afterpulsing probability can be reduced only by increasing the quencher dead-time, which may not be a good solution if high speed is a requirement. The potential for a significant reduction of the SPAD noise is critical in applications where the SPAD noise can deteriorate the performance. However, achieving the optimal operating conditions in terms of the gating characteristics (biasing level, duty cycle, and frequency) requires a careful characterization with respect to the operating condition to achieve a trade-off among noise, sensitivity (i.e., photon detection efficiency), and speed.

### 2.2. Time-Gated SPAD Detector Implementation

Figure 1b,c shows the circuit block diagram and the microphotograph of the fully integrated time-gated SPAD data receiver test chip, respectively [21]. The CMOS circuit part is supplied with $V_{\mathrm{DD}}=V_{\mathrm{SPAD}}=$ 3.3 V and $V_{\mathrm{SS}}=-$3.3 V and is isolated from the substrate, which forms the anode of the SPAD. Hence, the cathode–anode voltage of the SPAD can be adjusted to $V_{\mathrm{SPAD}}-V_{\mathrm{sub}}$ by applying a negative substrate voltage of $V_{\mathrm{sub}}$. The gating circuit is driven with a digital noninverted clock signal CLK, its inverted counterpart $\bar{\mathrm{CLK}}$, and a −3.3 V-level shifted clock $\bar{\mathrm{CLKD}}$. The cathode of the SPAD is switched between $V_{\mathrm{SPAD}}$ and $V_{\mathrm{SS}}$, resulting in a voltage swing of up to 6.6 V. During CLK $=V_{\mathrm{DD}}$, the cathode of the SPAD is charged up to ≈$V_{\mathrm{SPAD}}\approx$ $V_{\mathrm{DD}}=$ 3.3 V by turning on P0 at the beginning of the clock phase. After charge up, to be ready for detection, P0 is turned off. In the subsequent clock phase CLK = GND = 0 V, the cathode of the SPAD is pulled down to ≈−3.3 V by N0, regardless of whether in the previous phase an avalanche appeared or not. N2 helps with discharging the net PLS. To limit the drain–source voltage of transistors N0, P0, and P2 to ≈3.3 V, the cascode transistors N1 and P1 were added to divide the voltage drop of 6.6 V over two transistors. The biasing voltage on the SPAD (i.e., the cathode–anode voltage) is equal to $V_{\mathrm{SPAD}}$ − $V_{\mathrm{sub}}$ and $V_{\mathrm{SS}}$ − $V_{\mathrm{sub}}$ during the gate-ON and the gate-OFF times, respectively. This means the SPAD biasing voltage is gated between the voltage levels of $|\;V_{\mathrm{sub}}\;|\;$ − 3.3 V and $|\;V_{\mathrm{sub}}\;|\;$ + 3.3 V. The excess bias $V_{\mathrm{ex}}$ is the difference of the gate-ON biasing level ($|\;V_{\mathrm{sub}}\;|\;$ + 3.3 V) and the magnitude of the breakdown voltage of the SPAD. To be able to quench an avalanche, $V_{\mathrm{ex}}$ must be smaller than 6.6 V; otherwise, the SPAD biasing level will exceed the breakdown voltage during the gate-OFF time.

The duty cycle and clock frequency are defined by a clock signal, which is applied to the clock input pad of the chip. If during the detection phase, an avalanche occurs, the voltage at the CAT node decreases due to the avalanche current of the SPAD until the breakdown level is reached or node CAT is pulled down to $V_{\mathrm{SS}}$ in the subsequent clock phase, both of which quench the avalanche. Transmission gate P3, N4 is turned on during the active phase of the SPAD and turned off when the SPAD is switched off. Consequently, the voltage at node CAT at the end of the active phase of the SPAD is sampled and stored dynamically at node IN during CLK $=V_{\mathrm{SS}}$ at the negative input of the clocked comparator. The comparator itself is in reset mode during CLK $=V_{\mathrm{DD}}$, i.e., when the SPAD is active, and it compares the voltage level at node IN with $V_{\mathrm{ref}}$ during CLK $=V_{\mathrm{SS}}$, when the SPAD is switched off. In Goll et al. [22], transient measurements were performed with the same gating circuit, but with an off-chip SPAD of the same size (note that two bond pad capacitances and the capacitance of the probe needle had to be driven by the gating circuit in addition to the capacitance of the SPAD in Goll et al. [22]). The rise time of charging up the SPAD and the fall time for turning off the SPAD were less than 1 ns. This shows that the gating circuit is fast enough for active phase lengths of 5 ns (g= 0.1). When considering the absence of parasitic capacitances of two bond pads and a probe needle, the fully integrated version of Goll et al. [21], which was treated in this work as well, is even faster. This can be seen in Goll et al. [21], where the circuit was operated at 250 MHz.

It is worth mentioning that the voltage at node IN follows the cathode voltage of the SPAD in less than 100 ps according to the post-layout simulation. The SPAD itself, however, uses an epi layer to detect red light with higher efficiency as compared to a structure of a thin p+/n-well SPAD. On the other hand, the epi layer results in a reduction of the avalanche current and speed. Transient measurements on the cathode of off-chip SPADs of a similar type and size that were bonded to the same circuit (see Goll et al. [23]) revealed a fall time during avalanche of the cathode voltage to a breakdown level of approximately 10 ns, but this includes an additional load of two-times the parasitic capacitance of a pad and the input capacitance of the RF probe. Post-layout simulation shown an overall load capacitance of approximately 300 fF at the cathode of the on-chip SPAD in comparison to an estimated 700 fF load for the measured off-chip one.

### 2.3. Dark-Noise Statistics

The count rate in a dark condition is a good measure of the dark-count noise. We proposed to use the same measurement data to characterize afterpulsing defined as the chance to detect a detrapped carrier as it is irrelevant if the previous detection event has been triggered due to photon absorption or thermal generation of carries. This can avoid circuit or optical complications associated with the conventional afterpulsing characterization methods that use short optical pulses to fire avalanche events and then observe the afterpulse events after the initial photon-triggered avalanche.

As the dark-noise measurement is quite straightforward, the proposed method is much more efficient from the experimental point of view, and both noise mechanisms can be characterized based on the same data and using the analysis approaches that are presented in the following. It is important to note that when the dark-count rate is low (below $10^{3}$ cps range); the collection of enough data to perform accurate estimations may need a very long measurement time at room temperature. In such a case, we proposed to perform the experiment at higher temperatures as the dark-count rate increases exponentially with temperature and the count rate is dominated by the dark-count detections. It is obvious that performing the measurement at a higher temperature would result in obtaining a different set of SPAD performance parameters. Therefore, the SPAD performance parameters at a lower temperature can be obtained by extrapolation from the results obtained at higher temperatures. It should be mentioned that all the results here were obtained at room temperature, but the presented analysis approaches can be applied to the dark-noise data acquired at any temperature.

**Figure 2:**
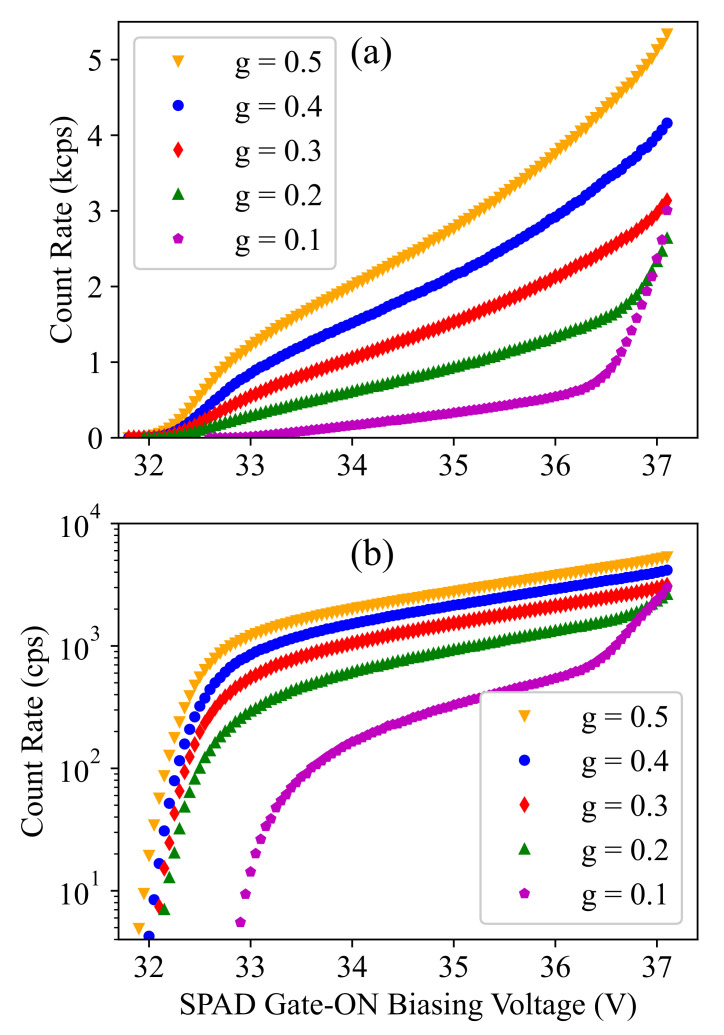
Measured detector count rate (in count per second) as a function of the SPAD gate-ON biasing voltage ($|\;V_{\mathrm{sub}}\;|\;$ + 3.3 V) and at different duty cycle (*g*) values plotted in linear (**a**) and logarithmic (**b**) scale. The values g={0.1,0.2,0.3,0.4,0.5} correspond to the gating-durations of $T_{G}=${5 ns, 10 ns, 15 ns, 20 ns, 25 ns} as the gating pulse rate is 20 MHz.

In order to obtain dark-noise data, a gating pulse with a frequency of 20 MHz ($T_{P}=50$ ns) and an amplitude of 6.6 V was applied to the SPAD cathode for 100 s (i.e., $2\times 10^{9}$ pulse periods). The pulse periods during which the detector counts an avalanche event were recorded, and this measurement was repeated at different operation conditions, i.e., different substrate (SPAD anode) voltage and pulse duty cycle ($g=T_{G}/T_{P}$) values. Figure 2a,b shows the number of recorded counts (per second) as a function of the gate-ON SPAD biasing voltage (i.e., the cathode–anode voltage given by $V_{\mathrm{SPAD}}-V_{\mathrm{sub}}$) at different *g* values with linear and logarithmic y-axis scales, respectively. Each data point on this plot corresponds to a specific operation condition in terms of biasing voltage and time-gating duty cycle (*g*). Please note that as we have $T_{P}=50$ ns, the equivalent gating-durations are given by $T_{G}=gT_{P}=g\times 50$ in ns units.

The measured count rate here gives a good sense of the dark-count rate except at very high biasing levels, where the count rate increases dramatically, as can be seen for g=0.1 (i.e., $T_{G}=0.1T_{P}=5$ ns). In such a condition, the detector cannot operate correctly; therefore, the biasing has to be kept below this limit. According to our investigation, the reason for such a large increase is a failure to fully recover (i.e., reaching a full-depletion of the device) after avalanche detections, which can be followed by tens or even hundreds of afterpulses. The reason that this dramatic increase appears as separate afterpulse sequences can be explained by the self-heating effect. In fact, the sequence continues until the chip heats up to a temperature where the SPAD breakdown voltage is increased to higher values at which the gating circuit can quench the SPAD completely during the gate-OFF time. Then, the chip starts to cool down, the breakdown voltage decreases, and the next train of detections starts again, and this loop continues, as was observed in our measurements.

The self-heating effect can also explain another observation here, that is the steep increase of the count rate is not seen at larger duty cycles for the same biasing condition. The reason is that due to the higher count rate at settings with a higher duty cycle, the local (junction) temperature of the die is higher compared to the case with a duty cycle of g=0.1. Although the PCB containing the quencher is mounted on a copper block, whose temperature is controlled to be 25 ${}^{{}^{\circ}}$C, a higher power dissipation results in a higher local temperature at the SPAD on the chip. The increased temperature at higher duty cycles results in a shift or, more accurately, a stretching of the measured dark-count characteristics towards higher biasing voltages. For a SPAD in the same technology, the temperature dependence of the DCR and the shift with respect to the substrate voltage were shown in Hofbauer et al. [24]. A change of temperature of 5 ${}^{{}^{\circ}}$C corresponds to a voltage shift of approximately 1 V for this type of SPAD. Consequently, we would see this steep increase further at the right for larger duty cycles in Figure 2. Please note that this breakdown shift (due to self-heating) is only apparent for larger excess bias voltages. For very low excess bias voltage, where we performed a breakdown calibration, explained later, the count rate is very low for all duty cycles and results in a negligible difference of the steady-state on-chip temperature. In the following sections, we restrict our SPAD characterization to applicable biasing conditions and describe how the data shown in Figure 2 can be used to characterize and model the breakdown voltage and the dark-count and afterpulsing noise of the SPAD.

## 3. Analysis and Modeling Approach

### 3.1. Gate-Duration Dependence of Effective Breakdown Voltage

The first important observation from Figure 2 is the voltage shift at the required biasing level for different duty cycle values. This shift is more clear in the plots with logarithmic y-axis (with a considerable value of around 1 V for g=0.1), and it demonstrates that the smaller the gating time $T_{G}$ is, the higher the required SPAD biasing voltage required to count avalanche events is. It is worth noticing that, when even the count rates at different duty cycles are normalize by (effective) $T_{G}$ (i.e., $T_{G}-T_{B}$ as is shown later), to allow a fair comparison based on normalized dark-count rates at different *g* values, the voltage shift remains almost the same. Therefore, this behavior cannot be explained by the “number of counts proportional to the effective $T_{G}$”, but it should be interpreted as an increase in the breakdown voltage for smaller duty cycles.

It is necessary to highlight that from a device physics point of view, the (theoretical) breakdown voltage, defined as the voltage where the multiplication factor of carriers approaches infinity [25], is a pure property of the SPAD device and cannot be affected by the characteristics (e.g., the sensitivity) of the frontend circuitry. However, from a higher level (system design) point of view for practical applications, the most important clue to measure and characterize the SPAD breakdown voltage is to identify the biasing level where the detector starts detecting. It is clear that based on this definition, the breakdown voltage cannot be independent of the features of the quenching/counting circuitry, i.e., we may measure a different breakdown for the same SPAD when a frontend circuitry of a different implementation or operation setting is used. Therefore, we need to characterize the “effective” breakdown behavior, which is affected by the properties of the frontend circuit and cannot be considered as a pure SPAD device parameter. Intuitively, unlike the free-running quench/reset operation where the avalanche charge can continue to generate a distinguishable amount of charge, in the time-gated operation, it can happen that during $T_{G}$, an avalanche fires, but before reaching the sensing threshold associated with the counting circuitry, the gate-ON time is over and the avalanche is suppressed before being detected.

From here on, the term “breakdown voltage” refers to an effective voltage value, which indicates a system-level performance indicator and shows a dependency on the gating duration, when the SPAD is operated in the time-gated mode. Accordingly, by calibrating the breakdown voltage, i.e., by excluding the $T_{G}$ dependence of the effective breakdown voltage from the total biasing on the SPAD, we present a unified expression of the SPAD noise (e.g., dark-count and afterpulsing) as a function of the excess bias, and only after this calibration, we can have the “number of counts proportional to the effective $T_{G}$” and it is possible to model the noise accordingly.

**Figure 3:**
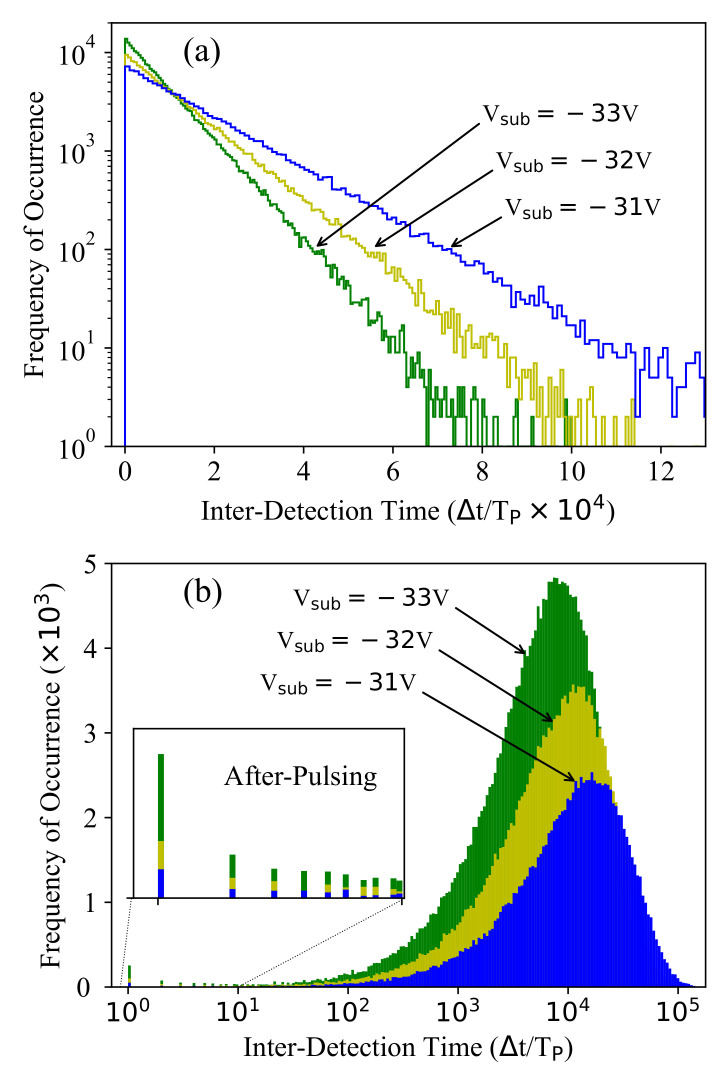
Distribution of the interdetection times at three different biasing conditions and g=0.2 with (**a**) uniform and (**b**) exponentially increasing bin width.

In order to provide a better understanding of the dark-noise measurement data indicated by the count rate in Figure 2, we investigated the distribution of the time intervals between successive counts as shown in Figure 3 for three different biasing conditions at g=0.2. In both histograms, the time bins are normalized by the gating pulse period, and therefore, both illustrate the distribution of the number of gated pulses between each two detected counts during a measurement running for 100 s (i.e., $2\times 10^{9}$ pulse periods).

Figure 3a shows the common representation where the time bins have an equal width (i.e., linear x-axis). This plot captures only the distribution corresponding to the dark-count process, which is known to be exponential [9]. As expected, a higher biasing results in a higher dark-count rate, which means a shorter average time interval between counts. Such a representation, however, ignores potentially valuable information about afterpulsing as the dark-noise measurement is dominated by the dark-counts and only a small fraction of the total counts are followed by afterpulse-counts. More importantly, afterpulsing exhibits a much shorter average time interval between counts, which cannot be characterized with time bins of a uniform width. Therefore, we preferred a second representation, shown in Figure 3b, where the bin width increases exponentially. In fact, as the detection probability of both noise mechanisms shows an exponential behavior with time and their average time intervals differ by several orders of magnitude, the distribution associated with the two noise mechanisms can be distinguished in this representation. Here, the average time interval between the dark-counts is around $10^{4}$ pulse periods ($T_{P}=50$ ns), while the afterpulse-counts show an average interdetection time of one to two pulse periods.

**Figure 4:**
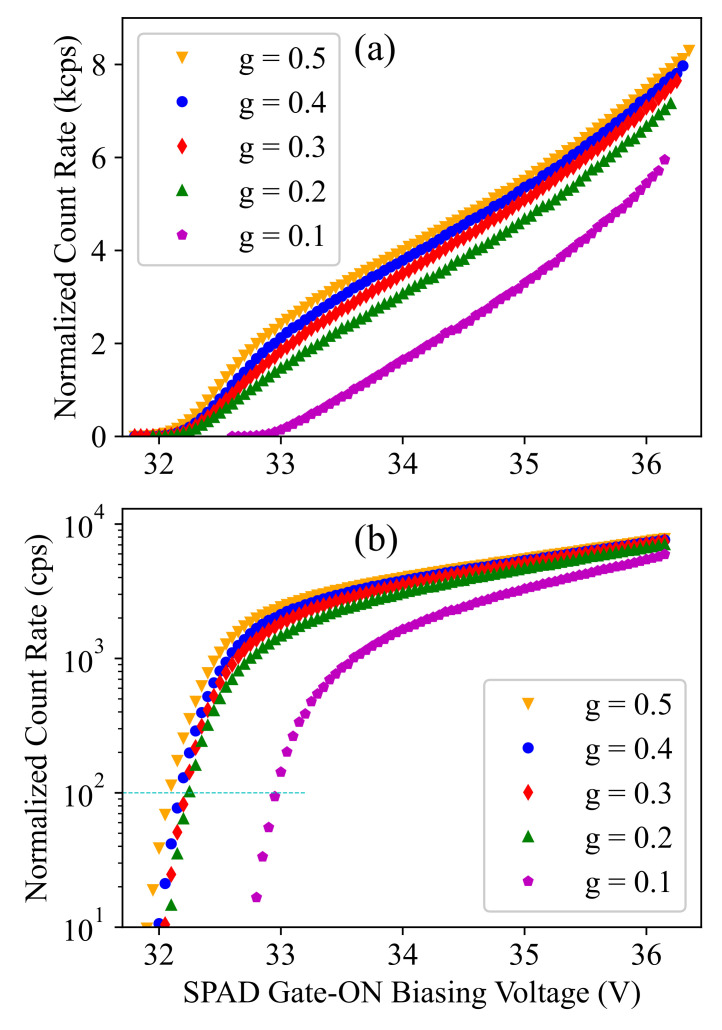
Dark-count rate as a function of the SPAD gate-ON biasing voltage ($|\;V_{\mathrm{sub}}\;|\;$ + 3.3 V) and at different duty cycle (*g*) values plotted in linear (**a**) and logarithmic (**b**) scale.

**Figure 5:**
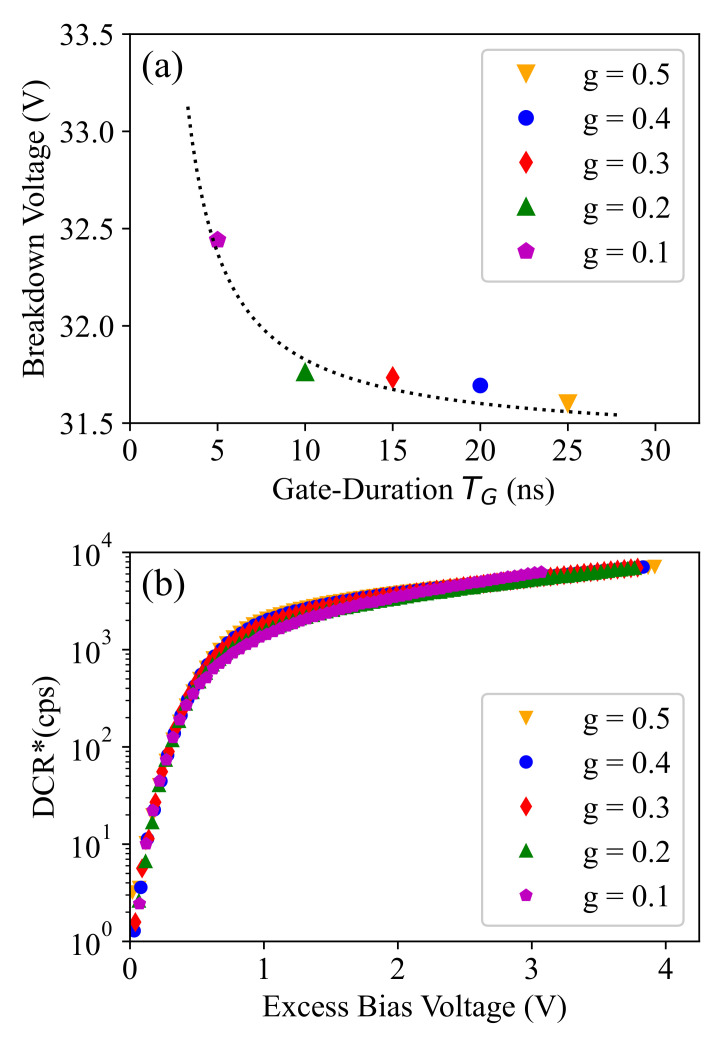
(**a**) Calibrated breakdown voltage as a function of the gate-ON time. (**b**) Total (maximum) dark-count rate after breakdown calibration as a function of the excess bias voltage at different *g* values.

Now, if we calculate the dark-count rate as $1/\bar{\Delta t}$, where $\bar{\Delta t}$ is the average time interval between the dark-counts, and normalize it by *g*, we expect to obtain similar count rates at each voltage biasing and independent of *g*, as it should correspond to the total (i.e., maximum) dark-count rate that can be obtained when g=1. However, the measurement results shown in Figure 4 do not follow this expectation, especially at smaller *g* values and at lower biasing, i.e., closer to the breakdown limit, as is highlighted in Figure 4b. This illustrates that the breakdown voltage shows a gating-duration dependence, and at each *g* value, we can calculate the breakdown shift by interpolating the biasing values corresponding to a specific count rate at a low rate value, e.g., between 10 and $10^{2}$, as is shown in Figure 4b. The obtained biasing values are shown in Figure 5a, and if we apply this breakdown calibration to the the total dark-count rate plotted as a function of the excess bias voltage, we obtain Figure 5b. Here, we achieved similar behavior regarding the maximum dark-count rate estimated based on the measured results at different *g* values.

It is important to note that when an avalanche process starts, the number of created carriers will have a near-exponential growth as each accelerated carrier can generate at least two more carriers (i.e., an electron–hole pair) via the impact–ionization mechanism. This initial exponential growth can happen in a very short time (sub-ns or ps range) depending on the SPAD size and the excess bias voltage. Then, the avalanche process can reach a (mature) self-sustaining condition, and if the device biasing is kept above a breakdown limit for a long enough time (in the ns range), the avalanche process can generate the minimum amount of charge needed to meet the sensing threshold of the counting circuitry. It is clear that, if the total avalanche duration time is shorter, as is the case for the time-gated operation with smaller $T_{G}$ settings, a higher biasing voltage is required to generate the same amount of charge. Therefore, at any $T_{G}$ setting, there is a minimum biasing voltage (denoted by $V_{\mathrm{BD}}\left(T_{G}\right)$) below which the detector will count any detection. If we define $V_{\mathrm{BD}0}$ as the absolute minimum breakdown, i.e., there is no detection even for very long $T_{G}$ values, a breakdown increase corresponding to a limited $T_{G}$ can be obtained as $\Delta V_{\mathrm{BD}}=V_{\mathrm{BD}}-V_{\mathrm{BD}0}$. We also define $T_{G0}$ as the extreme limit for the gate-duration time, i.e., if $T_{G}<T_{G0}$, the gate-ON time is too short such that the avalanche can generate a detectable amount of charge even if $V_{\mathrm{BD}}$ is set to a very high value. The effect of the limited speed of that gating signal is reflected by the parameter $T_{G0}$, and it includes the (subnanosecond) fall/rise time of the gating pulse. To enable the detector to count the avalanche events, $\Delta T_{G}=T_{G}-T_{G0}$ must be greater than zero. It is clear that the shorter the $\Delta T_{G}$, the higher the $\Delta V_{\mathrm{BD}}$. In order to model the relationship between $\Delta V_{\mathrm{BD}}$ and $\Delta T_{G}$, we used Equation (1), as it showed a good agreement with the results obtained by the interpolation approach that was explained before. Based on this simple model, the product of $\Delta V_{\mathrm{BD}}$ and $\Delta T_{G}$ is a constant value giving a measure proportional to a specific amount of charge (e.g., $Q_{0}$) that needs to be generated during the avalanche process to meet the detection threshold of the counter circuitry. This implicitly assumes a linear relationship between the (average) current generated during the avalanche process and $\Delta V_{\mathrm{BD}}$, and therefore, if, for example, $\Delta T_{G}$ is decreased by a factor of two, $\Delta V_{\mathrm{BD}}$ has to be increased by the same factor to provide $Q_{0}$.

(1) $$\Delta V_{\mathrm{BD}}\left(T_{G}\right)=V_{\mathrm{BD}}\left(T_{G}\right)-V_{\mathrm{BD}0}=\frac{B_{0}}{T_{G}-T_{G0}}.$$

Here, B0, $V_{\mathrm{BD}0}$, and $T_{G0}$ are (constant) model parameters and should be calibrated using the measurement data, and they were obtained as $B_{0}=3.8$ V.ns, $T_{G0}=1.1$ ns, and $V_{\mathrm{BD}0}=31.45$ V by fitting the model to the measurement results shown in Figure 5b. One should note that this model neglects the fact that an avalanche may trigger at any instant (e.g., $t_{0}$) during $T_{G}$, and if $t_{0}$ is closer to the falling edge of the gated pulse, the counter may not be able to detect the event even if the biasing is above the breakdown voltage corresponding to $T_{G}$. In fact, for the breakdown calibration, it is more reasonable to assume that the avalanche events are triggered at the beginning of $T_{G}$ as the interpolation was based on very low dark-count rates (e.g., $10^{2}$) in the DCR plot shown in Figure 4b. At such low rates, where the biasing voltage is slightly above the breakdown, only avalanche events that are triggered at the beginning of $T_{G}$ have the chance to be counted. In other words, the (low) dark-count rate, which is recorded at low excess bias voltages, is associated with the detections at the beginning of $T_{G}$, and the (thermally) generated carriers that could initiate an avalanche closer to the falling edge of the gated pulse are not counted as the excess bias is small and the avalanche process cannot generate enough charge to be detected. Consequently, the model provides a reasonable accuracy to calibrate the breakdown voltage as a function of $T_{G}$, and we took this $t_{0}$-dependent detection probability effect into account in the noise characterization and modeling, as will be explained latter. According to this model, the breakdown voltage as a function of $T_{G}$ can be obtained by:

(2) $$V_{\mathrm{BD}}\left(T_{G}\right)=V_{\mathrm{BD}0}+\frac{B_{0}}{T_{G}-T_{G0}}=31.45+\frac{3.8}{T_{G}-1.1}\;\left(V\right)$$

where $T_{G}$ is in ns and must be larger than $T_{G0}=1.1$ ns. It should be noted that the model can capture different properties or settings of the gating circuit via the constant model parameters. To be more specific, the parameters $T_{G0}$ can capture the (nonzero) rise time of the gating signal on the SPAD, and the parameter $B_{0}$ stands for the sensing threshold of the circuit, i.e., the comparator performance and the $V_{\mathrm{ref}}$ voltage setting. Furthermore, the model can be extended to include the temperature dependence of the breakdown voltage by expressing $V_{\mathrm{BD}0}$ as a function of temperature. However, as this is a well-known effect, we kept the equation simple, focusing on the gating-duration dependence of the SPAD breakdown.

### 3.2. Dark-Count Rate

The interdetection time between dark-counts has an exponential distribution with a time constant ($\tau_{\mathrm{dc}}$), which is equal to the average interdetection time. Furthermore, due to the memoryless nature of any process with an exponential distribution, not only the waiting time between the events, but also the waiting time between any random instant and the next upcoming (dark-count) event follows the same distribution with $\tau_{\mathrm{dc}}$, as was discussed in more detail in Mahmoudi et al. [9]. However, when the dark-count detection probability is studied over a time period (e.g., $T_{P}$) that is much shorter than the dark-count time constant ($T_{P}\ll \tau_{\mathrm{dc}}=1/DCR$), it can be shown that the probability of having a dark-count detection within $T_{P}$ can be obtained by $T_{P}/\tau_{\mathrm{dc}}$ with a good approximation. In our measurements, $\tau_{\mathrm{dc}}$ and $T_{P}$ are in the milli- and nanosecond ranges, respectively, and therefore, we can assume a linear relationship between $T_{P}$ and the corresponding dark-count probability.

**Figure 6:**
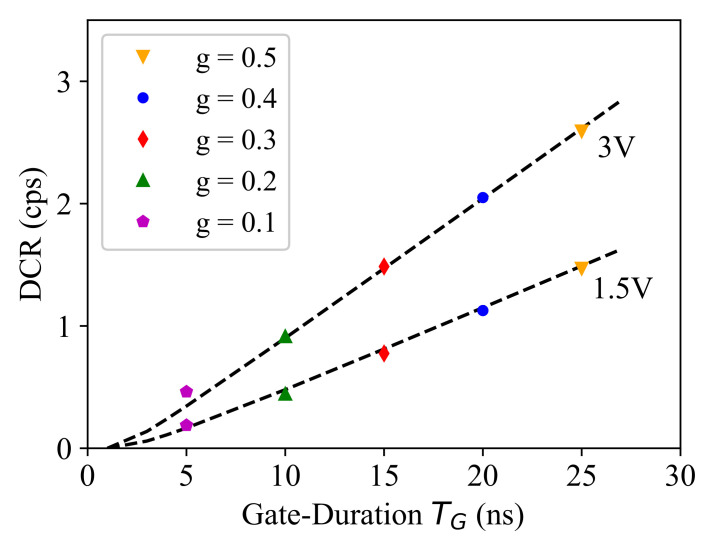
Dark-count rate as a function of the gate-ON time. The dashed lines show the model fit using Equation (4).

Accordingly, the measured dark-count rate must show a linear relationship with the SPAD active time, i.e., the gating duty cycle *g*. The raw measurement results, however, do not meet this expectation, as can be seen in Figure 4, and only after applying the $T_{G}$-dependent breakdown calibration, we obtained the result shown in Figure 6, where the dark-count rate shows a linear growth with $T_{G}$ at any specific excess bias voltage level. An observation here is that, the linear fit shown by the dashed line in Figure 6 crosses the *x*-axis at a nonzero $V_{\mathrm{ex}}$-dependent time value (denoted by $T_{B}$). We believe that this is a (blind) time corresponding to a fraction of the $T_{G}$, before its falling edge, where the gating circuit is still on, but blind to any detection. This is due to the same mechanism that was explained and modeled for breakdown calibration by Equation (1). In fact, if we consider the distribution of the avalanche triggering instant ($t_{0}$) over $T_{G}$, there is a time interval ($T_{B}$) at the end of the pulse period $T_{G}$, where the avalanche cannot grow enough to meet the sensing threshold of the counter. It is clear that the higher the $V_{\mathrm{ex}}$ is, the shorter the blind time $T_{B}$ is. Interestingly, $T_{B}$ can be estimated using the same model and the same model parameter described by Equation (1). In fact, we can assume $V_{\mathrm{ex}}$ as an extra breakdown shift corresponding to a shorter time interval $T_{B}<T_{G}$, and accordingly, if we replace $\Delta V_{\mathrm{BD}}$ and $T_{G}$ by $V_{\mathrm{ex}}$ and $T_{B}$, in Equation (1) respectively, we obtain:

(3) $$T_{B}\left(V_{\mathrm{ex}}\right)=T_{G0}+\frac{B_{0}}{V_{\mathrm{ex}}+B_{0}/\left(T_{G}-T_{G0}\right)}$$

where $T_{B}$ corresponds to the blind time within the gate-duration time $T_{G}$ when the gate excess bias is equal to $V_{\mathrm{ex}}$.

This correction allows us to accurately estimate the dark-count rate of the time-gated SPAD at as a function of $T_{G}$ by:

(4) $$DCR\left(T_{G},V_{\mathrm{ex}}\right)=DCR^{*}\left(V_{\mathrm{ex}}\right)\frac{T_{G}-T_{B}}{T_{P}-T_{B}}$$

**Figure 7:**
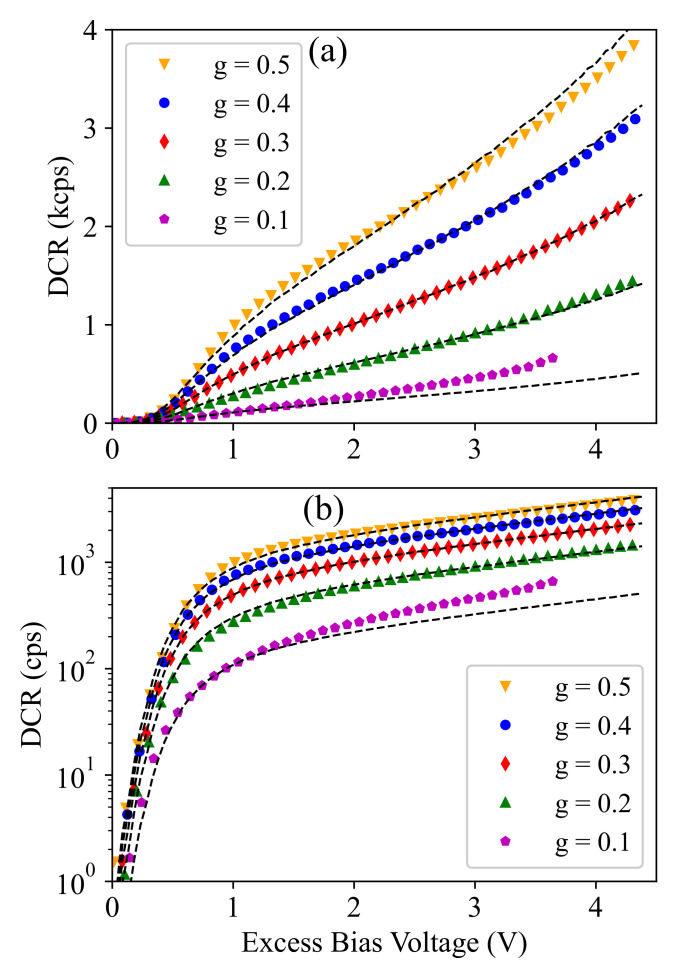
Dark-count rate as a function of the excess bias voltage at different duty cycle (*g*) values plotted in linear (**a**) and logarithmic (**b**) scale. The dashed lines show the model fit using Equation (4).

Here, $DCR^{*}$ is defined as the maximum possible dark-count rate corresponding to g=1 (i.e., $T_{G}=T_{P}$) at any specific $V_{\mathrm{ex}}$. This parameter can be calibrated according to the measurement results (at a given *g*) and then applied to estimate the dark-count rate at other *g* values. For example, Figure 7 compares the measurement results with the estimated DCR using Equation (4) (shown by dashed lines), where $DCR^{*}$ is calibrated according to the measurement results at g=0.3. This demonstrates that the model provides an accurate description and can be used for detector performance modeling and optimization to avoid time-consuming experimental optimization. In the next section, it is explained how the afterpulsing noise mechanism of SPAD can be modeled to cover both essential SPAD noise mechanisms required for performance modeling of a time-gated SPAD.

### 3.3. Afterpulsing Probability

Reducing the afterpulsing noise is a key motivation to operate the SPAD in time-gated mode. In fact, on the one hand, the number of filled traps is reduced as the flow time of the avalanche current is limited to the gate-ON time, and on the other hand, the afterpulsing probability regarding the filled traps is reduced as, during the gate-OFF time, the trapped carriers can be released without causing a detection count. This significantly reduces the afterpulsing; however, there is a trade-off between noise reduction and the photon detection efficiency of the detector due to the fact that a shorter gate-ON time means less chance for photon counting (assuming an asynchronous light source). Another parameter that imposes a similar trade-off is the excess bias voltage, as a higher excess bias voltage corresponds to a higher noise and a higher photon detection efficiency. The trade-off is more complicated when we need to include system-level parameters such as the data rate or counting decision threshold if one data bit contains more than one gated pulse [21]. Tuning several parameters to achieve an optimum detector performance may need extensive experimental or even redesign efforts. Therefore, accurate noise and performance modeling is necessary to reduce or avoid such efforts.

**Figure 8:**
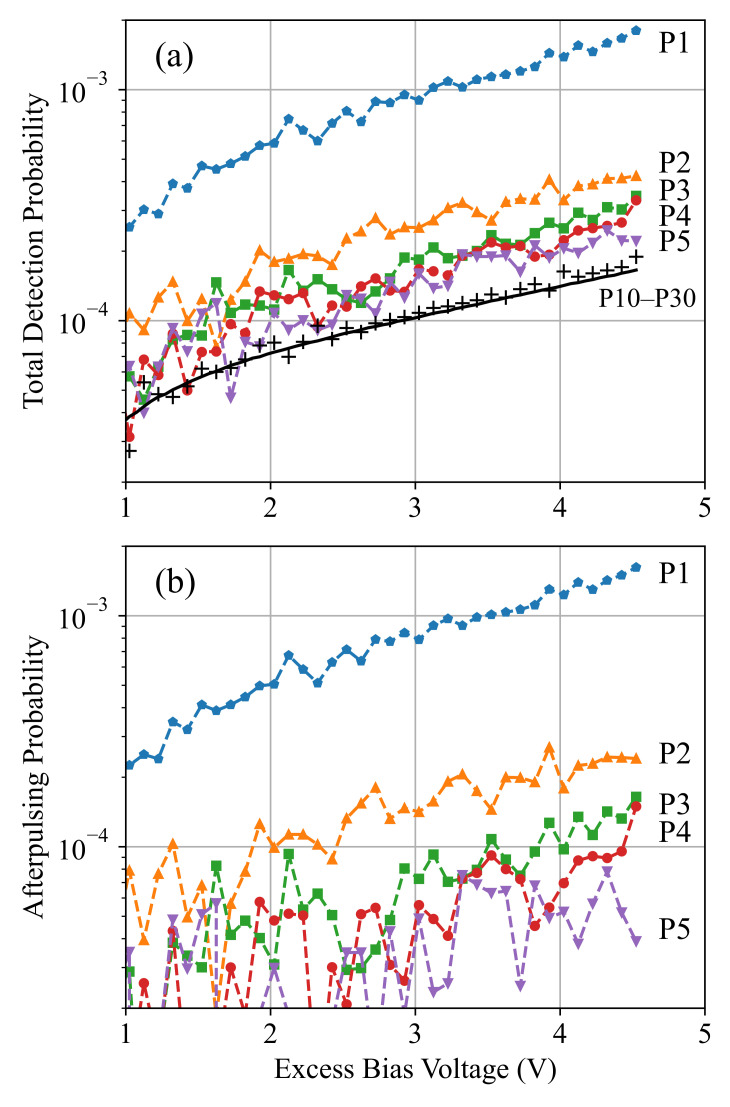
Total (**a**) and pure afterpulsing (**b**) detection probabilities in the first five pulse periods (P1–P5) after an avalanche detection as a function of $V_{\mathrm{ex}}$ at g=0.3. The (pure) afterpulsing probabilities are obtained by subtracting the estimated dark-count probabilities (shown by P10–P30) from the total (measured) detection probabilities.

In order to distinguish the afterpulsing from the dark-noise measurement data, we used the concept illustrated in Figure 3b, where the afterpulsing is associated with small interdetection times and shows the highest probability for the smallest interdetection time corresponding to one pulse period. Figure 8a shows the total detection probability for the first five bins (i.e., interdetections corresponding to $T_{P}$, 2$T_{P}$, *…*, 5$T_{P}$ denoted as P1–P5), obtained by dividing the number of counts in these bins by the total counts. The obtained value includes both afterpulsing and dark-count detections, and therefore, we should exclude the dark-count probability to obtain the pure afterpulsing probability (Figure 8b). The corresponding dark-count probability can be estimated either using the measurement data or using Equation (4). In fact, by averaging the measured detection probabilities of some bins with a negligible afterpulsing probability (e.g., time bins with interdetections of 10$T_{P}$ to 30$T_{P}$), we obtain the dark-count probability (shown by “+” for P10–P30 in Figure 8a) that must be excluded from P1–P5. This provides a similar result to the model estimation (shown by the solid line for P10–P30 in Figure 8a), and by applying this correction, we obtain the pure afterpulsing probabilities for P1–P5, as is shown in Figure 8b. This figure demonstrates that the afterpulsing shows the highest probability for P1, which indicates an interdetection of one pulse period $T_{P}$ and decreases exponentially with the interdetection time. This implicitly shows us that unlike the dark-count mechanism where we can assume a uniform detection probability distribution over $T_{P}$, a more accurate estimation of the afterpulsing probability distribution is required.

Although the SPAD device may have different types of trap at various energy levels having different release time behavior from the subnanosecond to above microsecond range, we are interested in the characterization of those dominating the nanosecond range, where the detector performance suffers the most. In fact, the faster traps (of the subnanosecond or a few ns range) are released during the gate-OFF time and before the next upcoming gated pulse. Furthermore, the slower traps are difficult to characterize as they mix up with dark-count detections (or background light noise in application mode), and most probably, in many applications it is not necessary to have a specific characterization or modeling for them, as they can be counted with other noise mechanisms. Therefore, in order to model the afterpulsing of the time-gated SPAD as a function of $T_{G}$, we assumed that the traps show a release time following an exponential distribution with a time constant of $\tau_{\mathrm{ap}}$ in the range of several nanoseconds. This is a reasonable assumption [9] and showed a very good agreement with the measurement data, as we will see in the following.

According to this assumption, if an avalanche is triggered at time $t_{0}$, the afterpulsing probability within the time interval ($t_{1},t_{2}$) after $t_{0}$ ($t_{1},t_{2}>t_{0}$) is obtained by:

(5) $$APP=APP^{*}\int_{t_{1}}^{t_{2}}\frac{1}{\tau_{\mathrm{ap}}}e^{-t/\tau_{\mathrm{ap}}}dt=APP^{*}\left(e^{-t_{1}/\tau_{\mathrm{ap}}}-e^{-t_{2}/\tau_{\mathrm{ap}}}\right),$$

where $APP^{*}$ is the total afterpulsing probability corresponding to ($t_{1}=t_{0}$ and $t_{2}=\infty$). It is clear that the SPAD was assumed to be active and ready to count between $t_{1}$ and $t_{2}$ with a biasing condition above the breakdown.

In the time-gated operation mode, we can assume that if an avalanche is triggered within $T_{G}$, the current flow through the SPAD (filling the traps) will continue over the whole gate-ON time, and therefore, the time that elapses between a detection and the next gate-ON pulse is equal to one gate-OFF period (i.e., $T_{P}-T_{G}$). The trapped carriers that are released during this period cannot trigger an afterpulse event. Then, when the SPAD is active again, there is a time period of $T_{G}-T_{B}$ during which an afterpulse avalanche can happen. This assumption provides a good approximation, especially at higher pulse rates, but as a secondary effect, one may take the transient behavior of the avalanche [22] into account to estimate the average (detection-free) time between an avalanche detection and the next gate-ON pulse more accurately.

Here, in order to obtain the afterpulsing probability corresponding to the *n*-th pulse after the avalanche detection (denoted by $APP(p_{n})$), we replaced $t_{1}$ and $t_{2}$ by $nT_{P}-T_{G}$ and $nT_{P}-T_{B}$ in Equation (5), respectively. As a result, the total afterpulsing probability of the time-gated SPAD can be obtained as:

(6) $$\begin{array}{cc} APP(T_{G},V_{\mathrm{ex}}) & =APP\left(p_{1}\right)+APP\left(p_{2}\right)+\ldots \\ \; & =APP^{*}\left(V_{\mathrm{ex}}\right)\left(e^{-\frac{T_{P}-T_{G}}{\tau_{\mathrm{ap}}}}-e^{-\frac{T_{P}-T_{B}}{\tau_{\mathrm{ap}}}}+e^{-\frac{2T_{P}-T_{G}}{\tau_{\mathrm{ap}}}}-e^{-\frac{2T_{P}-T_{B}}{\tau_{\mathrm{ap}}}}+\ldots \right)\\ \; & =APP^{*}\left(V_{\mathrm{ex}}\right)\left(\frac{e^{T_{G}/\tau_{\mathrm{ap}}}-e^{T_{B}/\tau_{\mathrm{ap}}}}{e^{T_{P}/\tau_{\mathrm{ap}}}-1}\right) \end{array}$$

The model parameters $\tau_{\mathrm{ap}}$ and $APP^{*}\left(V_{\mathrm{ex}}\right)$ were calibrated based on limited dark-noise measurement data, and then, the model can be used to estimate the afterpulsing probability at different *g* and $V_{\mathrm{ex}}$ values for any simulation purpose. In order to calculate $\tau_{\mathrm{ap}}$, if we divide the measured APPs for interdetection times of one and two $T_{P}$ (shown by p1 and p2 in Figure 8b), we have:

(7) $$\frac{APP(p_{1})}{APP(p_{2})}=\frac{e^{-\frac{T_{P}-T_{G}}{\tau_{\mathrm{ap}}}}-e^{-\frac{T_{P}-T_{B}}{\tau_{\mathrm{ap}}}}}{e^{-\frac{2T_{P}-T_{G}}{\tau_{\mathrm{ap}}}}-e^{-\frac{2T_{P}-T_{B}}{\tau_{\mathrm{ap}}}}}=e^{T_{P}/\tau_{\mathrm{ap}}}$$

As a result, $\tau_{\mathrm{ap}}$ can be obtained as:

(8) $$\tau_{\mathrm{ap}}=\frac{T_{P}}{\mathrm{Ln}\left(\frac{APP(p_{1})}{APP(p_{2})}\right)}$$

For our time-gated SPAD device in 0.35-μm CMOS technology, $\tau_{\mathrm{ap}}$ was obtained as around 26 ns, and this value provided an agreement between the model estimation and the measurement results regarding the afterpulsing probabilities at different gate-ON time values. One should note that if the dark-count probability is not excluded from the measurement result (as is shown in Figure 8a,b), $\tau_{\mathrm{ap}}$ cannot be captured accurately due to an overestimation in Equation (8) as both $APP(p_{1})$ and $APP(p_{2})$ will increase by a fixed amount equal to the dark-count probability within one pulse period. The other model parameter $APP^{*}\left(V_{\mathrm{ex}}\right)$ is defined as the afterpulsing probability when $T_{G}\to T_{P}\gg T_{B}$. That means that $APP^{*}\left(V_{\mathrm{ex}}\right)$ corresponds to the maximum possible afterpulsing probability at $V_{\mathrm{ex}}$, i.e., when the gate-OFF time and the blind time are negligible as compared to $T_{G}$. After $\tau_{\mathrm{ap}}$ is calculated and $APP(T_{G},V_{\mathrm{ex}})$ are obtained based on the measurement results at a specific $T_{G}$, the parameter $APP^{*}\left(V_{\mathrm{ex}}\right)$ can be calculated using Equation (6), and the model can be used to predict the afterpulsing probability at any other operation condition.

**Figure 9:**
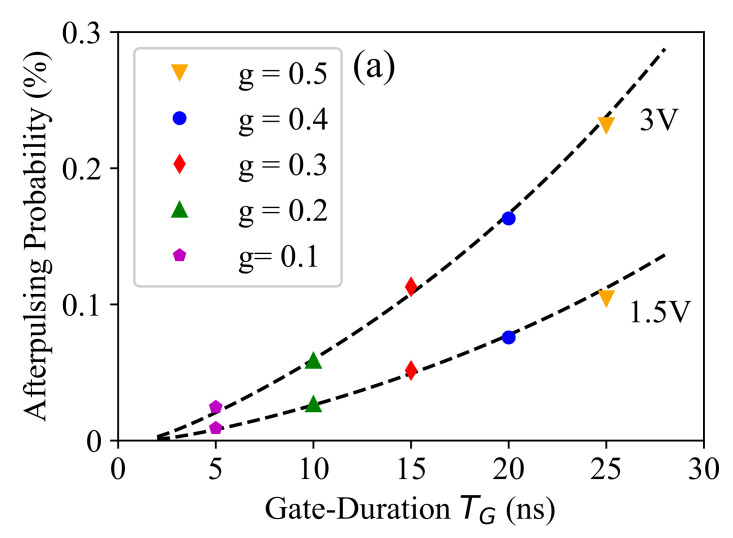

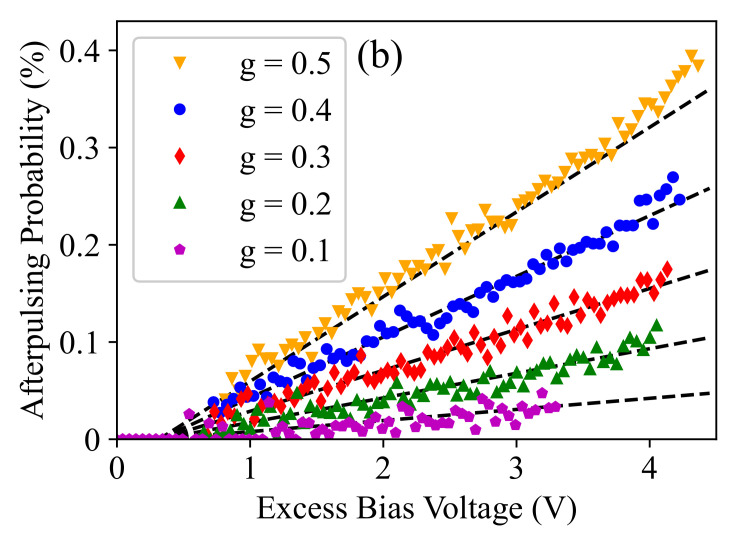
Afterpulsing probability as a function of the gate-ON time (**a**) and the excess bias voltage (**b**) at different duty cycle (*g*) values.

Figure 9a compares the measured afterpulsing probability (APP) and the corresponding estimated value using Equation (6) (shown by dashed lines) at different $T_{G}$ values. It demonstrates that the proposed afterpulsing probability model can accurately predict the exponential behavior of the afterpulsing probability as a function of $T_{G}$, which cannot be captured with a linear model used for dark-count characterization. Furthermore, Figure 9b shows the measurement and the model prediction (shown by dashed lines) results for the afterpulsing probability as a function of $V_{\mathrm{ex}}$ at different gated duty cycles. Here, the parameter $APP^{*}$ was calibrated at any $V_{\mathrm{ex}}$ value according to the measured value at g=0.3 and was used to predict the afterpulsing probability at other *g* values, and we saw a good agreement between the experimental data and the model prediction. It is interesting to note that here, the measurement data were more noisy as compared to that of the dark-count rate shown in Figure 7. The reason for the significant noise in the afterpulsing measurement data was the limited number of recorded events as compared to the dark-count events. In fact, as we characterized both noise mechanisms using the dark-noise measurement, the number of dark-count events was, by a factor of around 1/APP, larger than that of afterpulsing, which was about three orders of magnitude. It is clear that increasing the measurement time can decrease the measurement noise, but 100 s of measurement time at each operation condition provided a reasonable accuracy in our setup.

## 4. Discussion

**Figure 10:**
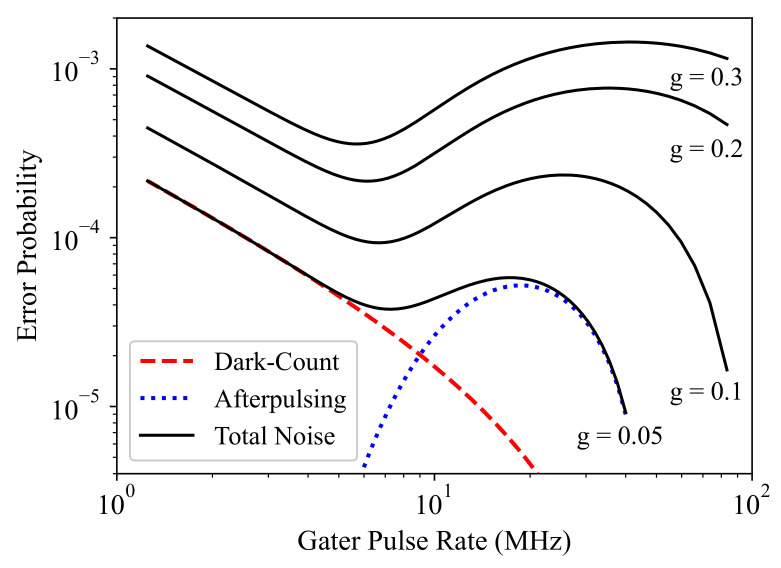
Noise detection probability as a function of the gated pulse rate at $V_{\mathrm{ex}}=3\;V$ and different *g* values.

In order to show the effectiveness of the proposed noise characterization and modeling approach, we calculated the error probability, i.e., counting an avalanche detection due to the SPAD intrinsic noise in a gated pulse, as a function of the pulse rate ($1/T_{P}$) to evaluate the detector noise behavior at different operation frequencies and to determine the optimum pulse rate with respect to noise. Figure 10 shows the obtained result for the dark-count and afterpulsing error probabilities ($E_{\mathrm{dc}}$ and $E_{\mathrm{ap}}$), as well as the total error probability calculated as $E_{\mathrm{dc}}+E_{\mathrm{ap}}-E_{\mathrm{dc}}E_{\mathrm{ap}}$ at $V_{\mathrm{ex}}=3\;V$ and different *g* values.

Here, $E_{\mathrm{dc}}$ showed a decrease with increasing pulse rate, and this was due to the fact that the shorter the gate-ON time, the smaller the dark-count detection probability in one gated pulse. $E_{\mathrm{ap}}$, however, showed a more complicated behavior with the pulse rate, as it was dominated by the gate-OFF time duration, during which the trapped carriers were released without causing an avalanche count. In fact, as the afterpulsing probability is majorly associated with the immediate pulse after the initial detection, an increase in the pulse rate shortens not only the gate-ON times, but also the time duration between them, where the latter dominates the afterpulsing probability. For pulse rates above a limit (around 20 MHz for g=0.05 in Figure 10), the afterpulsing probability starts to decrease with the frequency. This is due to the domination of the blind time $T_{B}$ effect as it becomes comparable with the gate-ON time $T_{G}$. This significantly affects not only the noise probability, but also the photon detection efficiency, and therefore, operating the detector at such pulse rates can seriously degrade the detector sensitivity. This may not be desirable when high sensitivity is a critical design parameter and may need a careful consideration. It is clear that both noise mechanisms and, thus, the total error probability decrease with *g*, as the shorter the gate-ON time is, the lower the chance for a noise count is. However, there arises a second trade-off between noise and sensitivity. It is interesting that there is a pulse frequency range where the total error (noise detection) probability is minimized (around 5–10 MHz), and this can be very attractive for a low-noise application of this detector.

This result provides great insight regarding the intrinsic noise behavior with respect to the gated pulse rate and shows the advantages of the proposed modeling approach, which needs only limited measurement data for calibration at a specific gated pulse rate, e.g., 20 MHz, which was used in our experimental studies. If such a modeling approach is not available, the experimental effort to explore the design space can increase exponentially with the number of design parameters that need to be evaluated and optimized.

## 5. Conclusions

Breakdown and noise characterization and modeling of SPAD detectors with time-gated operation was presented. The gating-duration dependence of the SPAD breakdown was introduced and modeled to capture the dark-count and afterpulsing probabilities with respect to excess bias voltage and gating duration time. The presented modeling approach requires limited dark-noise measurement data for calibration and can be used to better understand and optimize the SPAD-based detector designs and to avoid extensive experimental or even redesign efforts.

## Data Availability

The data presented in this study are available on request from the corresponding author.
